# Adamantan-1-aminium *p*-toluene­sulfonate

**DOI:** 10.1107/S1600536811003436

**Published:** 2011-02-02

**Authors:** Yi Zhang, Bo Wang

**Affiliations:** a Ordered Matter Science Research Center, Southeast University, Nanjing 211189, People’s Republic of China

## Abstract

There are two unique cations and anions in the asymmetric unit of the title mol­ecular salt, C_10_H_15_NH_3_^+^·C_7_H_7_O_3_S^−^. In the crystal, all three hydrogen-bond donors of the protonated amine group make hydrogen-bond inter­actions with sulfonate O-atom acceptors, linking the cations and anions into chains parallel to the *a* axis. C—H⋯π inter­actions are also present.

## Related literature

For related structures, see: Tukada & Mochizuki (2003 ▶); Zhao *et al.* (2003 ▶); Smith *et al.* (2004 ▶); He & Wen (2006 ▶); Zheng & Wang (2009 ▶). For puckering parameters, see: Cremer & Pople (1975 ▶). For ribbon hydrogen-bonding motifs, see: Hulme & Tocher (2006 ▶).
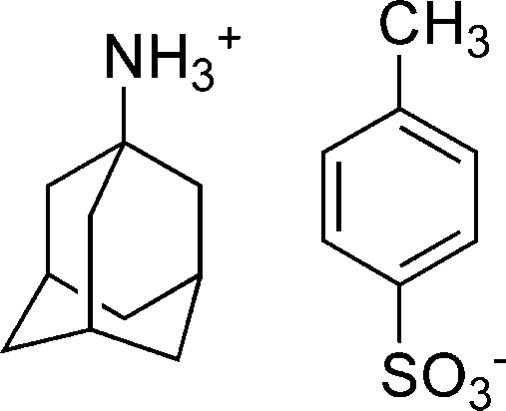


## Experimental

### 

#### Crystal data

C_10_H_18_N^+^·C_7_H_7_O_3_S^−^
*M* *_r_* = 323.44
Triclinic, 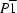

*a* = 6.464 (2) Å
*b* = 11.589 (4) Å
*c* = 22.562 (8) Å
α = 92.975 (4)°
β = 94.034 (5)°
γ = 96.408 (5)°
*V* = 1672.4 (10) Å^3^
*Z* = 4
Mo *K*α radiation
μ = 0.21 mm^−1^
*T* = 298 K
0.20 × 0.20 × 0.20 mm

#### Data collection

Rigaku SCXmini diffractometer
Absorption correction: multi-scan (*CrystalClear*; Rigaku, 2005 ▶) *T*_min_ = 0.960, *T*_max_ = 0.960
18425 measured reflections
7664 independent reflections
5720 reflections with *I* > 2σ(*I*)
*R*_int_ = 0.041

#### Refinement

*R*[*F* ^2^ > 2σ(*F* ^2^)] = 0.061
*wR*(*F* ^2^) = 0.149
*S* = 1.06
7664 reflections
421 parameters
6 restraints
H atoms treated by a mixture of independent and constrained refinement
Δρ_max_ = 0.45 e Å^−3^
Δρ_min_ = −0.36 e Å^−3^

### 

Data collection: *CrystalClear* (Rigaku, 2005 ▶); cell refinement: *CrystalClear*; data reduction: *CrystalClear*; program(s) used to solve structure: *SHELXS97* (Sheldrick, 2008 ▶); program(s) used to refine structure: *SHELXL97* (Sheldrick, 2008 ▶); molecular graphics: *SHELXTL* (Sheldrick, 2008 ▶); software used to prepare material for publication: *SHELXTL*.

## Supplementary Material

Crystal structure: contains datablocks I, global. DOI: 10.1107/S1600536811003436/jh2261sup1.cif

Structure factors: contains datablocks I. DOI: 10.1107/S1600536811003436/jh2261Isup2.hkl

Additional supplementary materials:  crystallographic information; 3D view; checkCIF report

## Figures and Tables

**Table 1:** Hydrogen-bond geometry (Å, °) *Cg*9 and *Cg*10 are the centroids of the C22–C27 and C29–C34 rings, respectively.

*D*—H⋯*A*	*D*—H	H⋯*A*	*D*⋯*A*	*D*—H⋯*A*
N1—H1*C*⋯O4	0.89 (2)	2.02 (2)	2.908 (3)	177 (3)
N1—H1*D*⋯O5^i^	0.90 (2)	1.99 (2)	2.883 (3)	177 (3)
N1—H1*E*⋯O6^ii^	0.89 (2)	1.92 (2)	2.806 (3)	173 (3)
N2—H2*C*⋯O1	0.91 (2)	1.93 (2)	2.834 (3)	174 (3)
N2—H2*B*⋯O2^iii^	0.89 (2)	1.92 (2)	2.806 (3)	170 (3)
N2—H2*A*⋯O3^iv^	0.89 (2)	2.01 (2)	2.901 (3)	175 (3)
C4—H4*A*⋯*Cg*10^iv^	0.98	3.18	3.878 (3)	130
C7—H7*B*⋯*Cg*9^iii^	0.97	2.87	3.801 (3)	161
C19—H19*B*⋯*Cg*10^v^	0.97	2.91	3.861 (3)	167

Symmetry codes: (i) 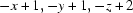; (ii) 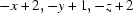; (iii) 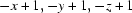; (iv) 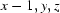; (v) 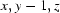.

**Fig. 1.**
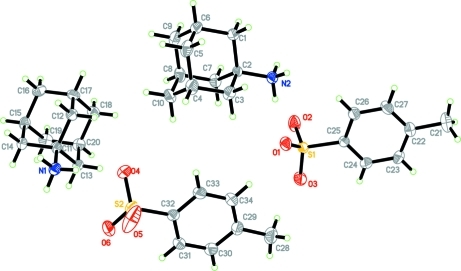
The molecular structure of the title compound, with the atomic numbering scheme and displacement ellipsoids drawn at the 30% probability level.

**Fig. 2.**
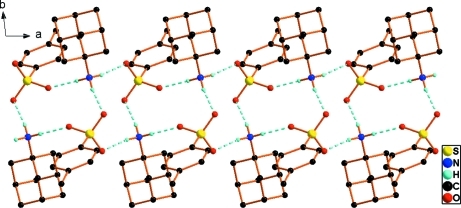
A view of the crystal packing of the title compound. Dashed lines indicate N–H···O hydrogen bonds which form infinite, one-dimensional chains along the a axis of the unit cell. H atoms not involved in hydrogen bonding have been omitted for clarity.

## supplementary crystallographic information

### Comment

Owing to its highly symmetrical and stable structure, adamantane and its derivatives have generated much interest in the past and continue to be actively studied as evidenced by the large number of compounds containing amantadine that have been synthesized (Tukada & Mochizuki, 2003; Zhao *et al.*, 2003; He & Wen, 2006). Our group have reported the crystal structures of the compounds of C_10_H_15_NH_3_^+^ .C_7_H_5_O_2_^-^. Here we report the synthesis and *CrystalStructure* of the title compound, (I), C_10_H_15_NH_3_^+^.C_7_H_7_O_3_S^-^, a salt obtained from the reaction of adamantane-1-ammonium hydrochloride and toluene-4-sulfonic acid sodium salt.

In the molecule of the title compound, the bond lengths and angles are within their normal ranges. There are two pairs of adamantane-1-ammonium cation and toluene-4-sulfonic acid anion in one asymmetric unit(Fig. 1). The dihedral angle between the benzene ring A (C22–C27) and benzene ring B (C29–C34) is A/B = 20.83 °. The two molecules are both stabilized by N—H···O hydrogen bonding, among which, N1—H1C···O4 and N2—H2C···O1 are intramolecular hydrogen bonds. All three hydrogen donors of the protonated amine group give direct hydrogen-bonding associations, with three of the sulfonate O-atom acceptors from three independent toluene-4-sulfonic acid anions. The hydrogen bonds are summarized in Tab. 1. Fig. 2 shows a view down the *c* axis. The N—H···O hydrogen bonds between the discrete adamantane-1-ammoniumcations and toluene-4-sulfonic acid anions result in a noteworthy one-dimensional ribbon-like structure parallel to (1 0 0) (Fig. 2). This ribbon motif is the dominant hydrogen-bonding motif (Hulme *et al.*, 2006). In addition, strong π-ring C7 –H7A···*Cg*9^iii^, C4 –H4B···*Cg*10^iv^, C19 –H19B··· *Cg*10^v^ interactions exist which contribute to crystal stability [*Cg*9 and *Cg*10 is the center of gravity of ring A and B, Symmetry code: (iii) -*x* + 1, -*y* + 1, -*z* + 1; (iv) *x* - 1, *y*, *z*; (v) *x*, *y* - 1, *z*.]

### Experimental

A mixture of adamantane-1-ammonium hydrochloride (10 mmol, 1.94 g), toluene-4-sulfonic acidsodium salt (10 mmol, 1.88 g) and methanol (50 ml) was stirred in a beaker. There were many solid powders produced and the solution was filtered. Colorless single crystals of the title compound suitable for X-ray analysis were obtained by slow evaporation of the solvents over a period of a week.

The dielectric constant of the compound as a function of temperature indicates that the permittivity is basically temperature-independent (*ε* = C/(T–T_0_)), suggesting that this compound is not ferroelectric or there may be no distinct phase transition occurring within the measured temperature range between 93 K and 362 K (m.p. 99 *^o^*C).

### Refinement

The positional parameters of all C-bound H atoms were calculated geometrically and allowed to ride, with *U*_iso_(H) = 1.5Ueq(C) for methyl H atoms and 1.2Ueq(C) for all other H atoms. All ammonium H atoms were found in a difference Fourier map and refined with restraints for the N—H distances of 0.87 (2) Å.

### Crystal data

**Table:**

C_10_H_18_N^+^·C_7_H_7_O_3_S^−^	*Z* = 4
*M**_r_* = 323.44	*F*(000) = 696
Triclinic, *P*1	*D*_x_ = 1.285 Mg m^−^^3^
Hall symbol: -P 1	Mo *K*α radiation, λ = 0.71073 Å
*a* = 6.464 (2) Å	Cell parameters from 2622 reflections
*b* = 11.589 (4) Å	θ = 3.0–27.5°
*c* = 22.562 (8) Å	µ = 0.21 mm^−^^1^
α = 92.975 (4)°	*T* = 298 K
β = 94.034 (5)°	Prism, colourless
γ = 96.408 (5)°	0.20 × 0.20 × 0.20 mm
*V* = 1672.4 (10) Å^3^	

### Data collection

**Table:**

Rigaku SCXmini diffractometer	7664 independent reflections
Radiation source: fine-focus sealed tube	5720 reflections with *I* > 2σ(*I*)
graphite	*R*_int_ = 0.041
Detector resolution: 13.6612 pixels mm^-1^	θ_max_ = 27.5°, θ_min_ = 3.2°
ω scans	*h* = −8→8
Absorption correction: multi-scan (*CrystalClear*; Rigaku, 2005)	*k* = −15→15
*T*_min_ = 0.960, *T*_max_ = 0.960	*l* = −29→29
18425 measured reflections	

### Refinement

**Table:**

Refinement on *F*^2^	Primary atom site location: structure-invariant direct methods
Least-squares matrix: full	Secondary atom site location: difference Fourier map
*R*[*F*^2^ > 2σ(*F*^2^)] = 0.061	Hydrogen site location: inferred from neighbouring sites
*wR*(*F*^2^) = 0.149	H atoms treated by a mixture of independent and constrained refinement
*S* = 1.06	*w* = 1/[σ^2^(*F*_o_^2^) + (0.0577*P*)^2^ + 0.5519*P*] where *P* = (*F*_o_^2^ + 2*F*_c_^2^)/3
7664 reflections	(Δ/σ)_max_ < 0.001
421 parameters	Δρ_max_ = 0.45 e Å^−^^3^
6 restraints	Δρ_min_ = −0.36 e Å^−^^3^

### Special details

**Table:**

Geometry. All e.s.d.'s (except the e.s.d. in the dihedral angle between two l.s. planes) are estimated using the full covariance matrix. The cell e.s.d.'s are taken into account individually in the estimation of e.s.d.'s in distances, angles and torsion angles; correlations between e.s.d.'s in cell parameters are only used when they are defined by crystal symmetry. An approximate (isotropic) treatment of cell e.s.d.'s is used for estimating e.s.d.'s involving l.s. planes.
Refinement. Refinement of *F*^2^ against ALL reflections. The weighted *R*-factor *wR* and goodness of fit *S* are based on *F*^2^, conventional *R*-factors *R* are based on *F*, with *F* set to zero for negative *F*^2^. The threshold expression of *F*^2^ > σ(*F*^2^) is used only for calculating *R*-factors(gt) *etc*. and is not relevant to the choice of reflections for refinement. *R*-factors based on *F*^2^ are statistically about twice as large as those based on *F*, and *R*- factors based on ALL data will be even larger.

### Fractional atomic coordinates and isotropic or equivalent isotropic displacement parameters (Å^2^)

**Table:**

	*x*	*y*	*z*	*U*_iso_*/*U*_eq_	
S1	0.77493 (9)	0.69083 (5)	0.55264 (3)	0.03980 (16)	
S2	0.74660 (10)	0.63466 (5)	0.92134 (3)	0.04414 (17)	
O1	0.6491 (3)	0.69428 (16)	0.60347 (7)	0.0496 (4)	
O2	0.7429 (4)	0.57650 (17)	0.52171 (8)	0.0690 (6)	
O3	0.9931 (3)	0.73164 (18)	0.56771 (9)	0.0624 (5)	
O4	0.6009 (3)	0.53539 (17)	0.90067 (9)	0.0704 (6)	
O5	0.6549 (5)	0.72161 (19)	0.95484 (12)	0.1036 (10)	
O6	0.9274 (3)	0.5982 (2)	0.95394 (9)	0.0757 (7)	
N1	0.6926 (4)	0.34970 (18)	0.97681 (10)	0.0404 (5)	
H1E	0.807 (3)	0.369 (2)	1.0008 (10)	0.051 (8)*	
H1D	0.584 (4)	0.331 (2)	0.9986 (11)	0.065 (9)*	
H1C	0.662 (4)	0.408 (2)	0.9545 (11)	0.066 (9)*	
N2	0.2620 (3)	0.5509 (2)	0.58768 (10)	0.0406 (5)	
H2C	0.390 (3)	0.593 (2)	0.5944 (12)	0.057 (8)*	
H2B	0.254 (5)	0.503 (2)	0.5550 (11)	0.077 (11)*	
H2A	0.172 (4)	0.603 (2)	0.5820 (12)	0.060 (9)*	
C1	−0.0056 (4)	0.4160 (2)	0.62903 (11)	0.0451 (6)	
H1A	−0.0101	0.3636	0.5939	0.054*	
H1B	−0.1082	0.4698	0.6221	0.054*	
C2	0.2118 (3)	0.48292 (19)	0.64076 (9)	0.0323 (5)	
C3	0.2180 (4)	0.5664 (2)	0.69513 (11)	0.0478 (6)	
H3A	0.1161	0.6208	0.6888	0.057*	
H3B	0.3552	0.6104	0.7019	0.057*	
C4	0.1690 (5)	0.4966 (2)	0.74942 (11)	0.0540 (7)	
H4A	0.1728	0.5500	0.7847	0.065*	
C5	−0.0484 (4)	0.4300 (3)	0.73803 (12)	0.0543 (7)	
H5A	−0.0827	0.3868	0.7724	0.065*	
H5B	−0.1507	0.4842	0.7318	0.065*	
C6	−0.0558 (4)	0.3464 (2)	0.68349 (12)	0.0487 (6)	
H6A	−0.1957	0.3035	0.6766	0.058*	
C7	0.3731 (4)	0.3978 (2)	0.65023 (11)	0.0455 (6)	
H7A	0.5118	0.4401	0.6569	0.055*	
H7B	0.3703	0.3455	0.6151	0.055*	
C8	0.3228 (4)	0.3284 (2)	0.70416 (12)	0.0505 (7)	
H8A	0.4257	0.2734	0.7105	0.061*	
C9	0.1040 (4)	0.2613 (2)	0.69326 (12)	0.0530 (7)	
H9A	0.0989	0.2077	0.6586	0.064*	
H9B	0.0721	0.2165	0.7273	0.064*	
C10	0.3292 (5)	0.4114 (3)	0.75947 (12)	0.0593 (8)	
H10A	0.2984	0.3675	0.7939	0.071*	
H10B	0.4676	0.4536	0.7670	0.071*	
C11	0.7115 (3)	0.24380 (18)	0.93733 (9)	0.0313 (5)	
C12	0.5136 (4)	0.2176 (2)	0.89602 (11)	0.0404 (5)	
H12A	0.4953	0.2834	0.8721	0.049*	
H12B	0.3934	0.2042	0.9192	0.049*	
C13	0.9013 (4)	0.2673 (2)	0.90126 (10)	0.0394 (5)	
H13A	1.0267	0.2854	0.9278	0.047*	
H13B	0.8857	0.3331	0.8772	0.047*	
C14	0.7379 (4)	0.14183 (19)	0.97613 (10)	0.0389 (5)	
H14A	0.6193	0.1286	0.9999	0.047*	
H14B	0.8631	0.1591	1.0028	0.047*	
C15	0.7543 (4)	0.0330 (2)	0.93597 (11)	0.0441 (6)	
H15A	0.7712	−0.0331	0.9605	0.053*	
C16	0.5573 (4)	0.0060 (2)	0.89403 (12)	0.0498 (6)	
H16A	0.4366	−0.0086	0.9170	0.060*	
H16B	0.5672	−0.0633	0.8688	0.060*	
C17	0.5313 (4)	0.1090 (2)	0.85546 (11)	0.0460 (6)	
H17A	0.4046	0.0916	0.8286	0.055*	
C18	0.7210 (4)	0.1309 (3)	0.81899 (11)	0.0539 (7)	
H18A	0.7045	0.1956	0.7941	0.065*	
H18B	0.7328	0.0625	0.7933	0.065*	
C19	0.9435 (4)	0.0554 (2)	0.89951 (13)	0.0514 (7)	
H19A	0.9563	−0.0133	0.8742	0.062*	
H19B	1.0694	0.0716	0.9260	0.062*	
C20	0.9185 (4)	0.1586 (2)	0.86126 (11)	0.0458 (6)	
H20A	1.0402	0.1723	0.8379	0.055*	
C21	0.4410 (6)	1.0064 (3)	0.37104 (14)	0.0785 (10)	
H21A	0.2931	0.9859	0.3635	0.118*	
H21B	0.5080	0.9951	0.3349	0.118*	
H21C	0.4687	1.0866	0.3853	0.118*	
C22	0.5244 (4)	0.9307 (2)	0.41724 (11)	0.0480 (6)	
C23	0.7334 (5)	0.9425 (2)	0.43641 (12)	0.0541 (7)	
H23A	0.8241	0.9993	0.4209	0.065*	
C24	0.8118 (4)	0.8718 (2)	0.47826 (11)	0.0463 (6)	
H24A	0.9535	0.8811	0.4904	0.056*	
C25	0.6786 (3)	0.7876 (2)	0.50179 (10)	0.0362 (5)	
C26	0.4681 (4)	0.7753 (3)	0.48367 (12)	0.0509 (7)	
H26A	0.3768	0.7196	0.4997	0.061*	
C27	0.3941 (4)	0.8462 (3)	0.44176 (12)	0.0525 (7)	
H27A	0.2524	0.8369	0.4296	0.063*	
C28	1.0559 (6)	0.8874 (3)	0.70974 (14)	0.0758 (10)	
H28A	1.2028	0.9116	0.7170	0.114*	
H28B	0.9827	0.9546	0.7059	0.114*	
H28C	1.0310	0.8386	0.6737	0.114*	
C29	0.9792 (4)	0.8206 (2)	0.76101 (11)	0.0474 (6)	
C30	1.1150 (4)	0.8022 (2)	0.80870 (11)	0.0460 (6)	
H30A	1.2555	0.8302	0.8084	0.055*	
C31	1.0482 (4)	0.7432 (2)	0.85693 (11)	0.0423 (6)	
H31A	1.1430	0.7314	0.8883	0.051*	
C32	0.8394 (4)	0.70212 (19)	0.85811 (10)	0.0361 (5)	
C33	0.7015 (4)	0.7182 (2)	0.81016 (11)	0.0479 (6)	
H33A	0.5612	0.6897	0.8103	0.057*	
C34	0.7716 (5)	0.7760 (2)	0.76237 (12)	0.0551 (7)	
H34A	0.6778	0.7854	0.7303	0.066*	

### Atomic displacement parameters (Å^2^)

**Table:**

	*U*^11^	*U*^22^	*U*^33^	*U*^12^	*U*^13^	*U*^23^
S1	0.0379 (3)	0.0447 (3)	0.0370 (3)	0.0042 (2)	0.0006 (2)	0.0077 (3)
S2	0.0530 (4)	0.0326 (3)	0.0478 (4)	0.0037 (3)	0.0118 (3)	0.0035 (3)
O1	0.0476 (10)	0.0645 (12)	0.0381 (9)	0.0044 (8)	0.0080 (8)	0.0134 (8)
O2	0.1153 (18)	0.0447 (11)	0.0475 (11)	0.0191 (11)	−0.0045 (11)	0.0012 (9)
O3	0.0337 (9)	0.0809 (14)	0.0734 (13)	0.0027 (9)	−0.0023 (9)	0.0287 (11)
O4	0.0761 (14)	0.0513 (12)	0.0760 (14)	−0.0211 (10)	−0.0186 (11)	0.0233 (10)
O5	0.163 (3)	0.0508 (13)	0.116 (2)	0.0313 (15)	0.1022 (19)	0.0185 (13)
O6	0.0668 (14)	0.0986 (17)	0.0568 (12)	−0.0103 (12)	−0.0185 (11)	0.0335 (12)
N1	0.0456 (13)	0.0327 (11)	0.0431 (12)	0.0036 (9)	0.0064 (10)	0.0025 (9)
N2	0.0378 (12)	0.0466 (13)	0.0380 (11)	0.0032 (10)	0.0046 (9)	0.0095 (10)
C1	0.0362 (13)	0.0532 (15)	0.0452 (14)	−0.0016 (11)	−0.0002 (11)	0.0142 (12)
C2	0.0287 (10)	0.0378 (12)	0.0310 (11)	0.0037 (9)	0.0027 (9)	0.0072 (9)
C3	0.0614 (16)	0.0399 (13)	0.0422 (14)	0.0020 (12)	0.0107 (12)	0.0011 (11)
C4	0.078 (2)	0.0509 (16)	0.0340 (13)	0.0054 (14)	0.0130 (13)	−0.0007 (12)
C5	0.0557 (16)	0.0606 (17)	0.0534 (16)	0.0161 (13)	0.0258 (13)	0.0188 (14)
C6	0.0381 (13)	0.0541 (16)	0.0537 (16)	−0.0042 (11)	0.0058 (12)	0.0182 (13)
C7	0.0385 (13)	0.0559 (16)	0.0464 (14)	0.0162 (11)	0.0094 (11)	0.0115 (12)
C8	0.0458 (14)	0.0590 (17)	0.0528 (15)	0.0242 (12)	0.0065 (12)	0.0204 (13)
C9	0.0717 (19)	0.0403 (14)	0.0479 (15)	0.0025 (13)	0.0110 (14)	0.0108 (12)
C10	0.0559 (17)	0.080 (2)	0.0396 (14)	−0.0022 (15)	−0.0075 (13)	0.0171 (14)
C11	0.0351 (11)	0.0266 (10)	0.0333 (11)	0.0054 (8)	0.0051 (9)	0.0043 (9)
C12	0.0346 (12)	0.0394 (13)	0.0485 (14)	0.0088 (10)	−0.0002 (10)	0.0082 (11)
C13	0.0358 (12)	0.0433 (13)	0.0392 (13)	−0.0007 (10)	0.0063 (10)	0.0082 (10)
C14	0.0436 (13)	0.0361 (12)	0.0382 (12)	0.0061 (10)	0.0050 (10)	0.0087 (10)
C15	0.0524 (15)	0.0320 (12)	0.0496 (14)	0.0094 (10)	0.0037 (12)	0.0091 (11)
C16	0.0497 (15)	0.0355 (13)	0.0623 (17)	−0.0018 (11)	0.0060 (13)	−0.0019 (12)
C17	0.0389 (13)	0.0485 (14)	0.0468 (14)	−0.0009 (11)	−0.0097 (11)	−0.0018 (12)
C18	0.0636 (17)	0.0579 (17)	0.0390 (14)	0.0056 (13)	0.0024 (13)	−0.0025 (12)
C19	0.0452 (14)	0.0496 (15)	0.0608 (17)	0.0163 (12)	0.0041 (13)	−0.0063 (13)
C20	0.0395 (13)	0.0551 (15)	0.0438 (14)	0.0054 (11)	0.0142 (11)	−0.0021 (12)
C21	0.097 (3)	0.084 (2)	0.063 (2)	0.038 (2)	0.0034 (19)	0.0289 (18)
C22	0.0590 (16)	0.0494 (15)	0.0385 (13)	0.0179 (12)	0.0036 (12)	0.0059 (11)
C23	0.0620 (18)	0.0457 (15)	0.0543 (16)	−0.0031 (13)	0.0072 (14)	0.0165 (13)
C24	0.0389 (13)	0.0460 (14)	0.0526 (15)	−0.0018 (11)	0.0024 (11)	0.0069 (12)
C25	0.0344 (12)	0.0399 (12)	0.0345 (12)	0.0038 (9)	0.0049 (9)	0.0030 (10)
C26	0.0361 (13)	0.0649 (17)	0.0526 (16)	0.0009 (12)	0.0050 (12)	0.0204 (13)
C27	0.0388 (14)	0.0727 (19)	0.0477 (15)	0.0118 (13)	0.0000 (12)	0.0122 (14)
C28	0.101 (3)	0.072 (2)	0.0554 (18)	0.0016 (19)	0.0131 (18)	0.0179 (17)
C29	0.0646 (17)	0.0393 (13)	0.0388 (13)	0.0066 (12)	0.0070 (12)	0.0013 (11)
C30	0.0437 (14)	0.0483 (15)	0.0456 (14)	0.0022 (11)	0.0079 (11)	−0.0009 (12)
C31	0.0402 (13)	0.0488 (14)	0.0375 (13)	0.0064 (11)	0.0011 (10)	−0.0012 (11)
C32	0.0403 (12)	0.0305 (11)	0.0371 (12)	0.0058 (9)	0.0023 (10)	−0.0037 (9)
C33	0.0411 (14)	0.0488 (15)	0.0520 (15)	0.0028 (11)	−0.0048 (12)	0.0030 (12)
C34	0.0592 (17)	0.0576 (17)	0.0460 (15)	0.0035 (13)	−0.0117 (13)	0.0078 (13)

### Geometric parameters (Å, °)

**Table:**

S1—O3	1.4474 (19)	C13—C20	1.530 (3)
S1—O2	1.452 (2)	C13—H13A	0.9700
S1—O1	1.4535 (18)	C13—H13B	0.9700
S1—C25	1.775 (2)	C14—C15	1.532 (3)
S2—O5	1.434 (2)	C14—H14A	0.9700
S2—O4	1.438 (2)	C14—H14B	0.9700
S2—O6	1.451 (2)	C15—C16	1.525 (4)
S2—C32	1.771 (2)	C15—C19	1.527 (4)
N1—C11	1.500 (3)	C15—H15A	0.9800
N1—H1E	0.886 (17)	C16—C17	1.530 (4)
N1—H1D	0.897 (17)	C16—H16A	0.9700
N1—H1C	0.894 (17)	C16—H16B	0.9700
N2—C2	1.502 (3)	C17—C18	1.530 (4)
N2—H2C	0.908 (17)	C17—H17A	0.9800
N2—H2B	0.894 (18)	C18—C20	1.532 (4)
N2—H2A	0.894 (17)	C18—H18A	0.9700
C1—C2	1.526 (3)	C18—H18B	0.9700
C1—C6	1.538 (3)	C19—C20	1.527 (4)
C1—H1A	0.9700	C19—H19A	0.9700
C1—H1B	0.9700	C19—H19B	0.9700
C2—C3	1.518 (3)	C20—H20A	0.9800
C2—C7	1.525 (3)	C21—C22	1.507 (4)
C3—C4	1.535 (3)	C21—H21A	0.9600
C3—H3A	0.9700	C21—H21B	0.9600
C3—H3B	0.9700	C21—H21C	0.9600
C4—C10	1.523 (4)	C22—C23	1.379 (4)
C4—C5	1.525 (4)	C22—C27	1.383 (4)
C4—H4A	0.9800	C23—C24	1.388 (4)
C5—C6	1.520 (4)	C23—H23A	0.9300
C5—H5A	0.9700	C24—C25	1.381 (3)
C5—H5B	0.9700	C24—H24A	0.9300
C6—C9	1.519 (4)	C25—C26	1.382 (3)
C6—H6A	0.9800	C26—C27	1.379 (4)
C7—C8	1.528 (3)	C26—H26A	0.9300
C7—H7A	0.9700	C27—H27A	0.9300
C7—H7B	0.9700	C28—C29	1.507 (4)
C8—C10	1.531 (4)	C28—H28A	0.9600
C8—C9	1.533 (4)	C28—H28B	0.9600
C8—H8A	0.9800	C28—H28C	0.9600
C9—H9A	0.9700	C29—C30	1.381 (4)
C9—H9B	0.9700	C29—C34	1.386 (4)
C10—H10A	0.9700	C30—C31	1.384 (3)
C10—H10B	0.9700	C30—H30A	0.9300
C11—C12	1.521 (3)	C31—C32	1.382 (3)
C11—C14	1.524 (3)	C31—H31A	0.9300
C11—C13	1.527 (3)	C32—C33	1.386 (3)
C12—C17	1.537 (3)	C33—C34	1.376 (4)
C12—H12A	0.9700	C33—H33A	0.9300
C12—H12B	0.9700	C34—H34A	0.9300
			
O3—S1—O2	113.03 (14)	C11—C13—C20	108.48 (19)
O3—S1—O1	113.08 (11)	C11—C13—H13A	110.0
O2—S1—O1	110.73 (13)	C20—C13—H13A	110.0
O3—S1—C25	106.86 (11)	C11—C13—H13B	110.0
O2—S1—C25	105.94 (11)	C20—C13—H13B	110.0
O1—S1—C25	106.65 (11)	H13A—C13—H13B	108.4
O5—S2—O4	113.53 (17)	C11—C14—C15	108.98 (18)
O5—S2—O6	111.87 (17)	C11—C14—H14A	109.9
O4—S2—O6	110.51 (13)	C15—C14—H14A	109.9
O5—S2—C32	106.22 (12)	C11—C14—H14B	109.9
O4—S2—C32	107.86 (12)	C15—C14—H14B	109.9
O6—S2—C32	106.43 (12)	H14A—C14—H14B	108.3
C11—N1—H1E	111.5 (18)	C16—C15—C19	109.4 (2)
C11—N1—H1D	105.9 (19)	C16—C15—C14	109.8 (2)
H1E—N1—H1D	109 (3)	C19—C15—C14	108.9 (2)
C11—N1—H1C	109.6 (19)	C16—C15—H15A	109.6
H1E—N1—H1C	112 (3)	C19—C15—H15A	109.6
H1D—N1—H1C	108 (3)	C14—C15—H15A	109.6
C2—N2—H2C	110.1 (17)	C15—C16—C17	109.6 (2)
C2—N2—H2B	110 (2)	C15—C16—H16A	109.8
H2C—N2—H2B	112 (3)	C17—C16—H16A	109.8
C2—N2—H2A	110.5 (18)	C15—C16—H16B	109.8
H2C—N2—H2A	106 (2)	C17—C16—H16B	109.8
H2B—N2—H2A	109 (3)	H16A—C16—H16B	108.2
C2—C1—C6	108.78 (19)	C18—C17—C16	109.5 (2)
C2—C1—H1A	109.9	C18—C17—C12	109.5 (2)
C6—C1—H1A	109.9	C16—C17—C12	109.1 (2)
C2—C1—H1B	109.9	C18—C17—H17A	109.6
C6—C1—H1B	109.9	C16—C17—H17A	109.6
H1A—C1—H1B	108.3	C12—C17—H17A	109.6
N2—C2—C3	109.12 (19)	C17—C18—C20	109.3 (2)
N2—C2—C7	108.77 (18)	C17—C18—H18A	109.8
C3—C2—C7	110.1 (2)	C20—C18—H18A	109.8
N2—C2—C1	109.05 (18)	C17—C18—H18B	109.8
C3—C2—C1	110.0 (2)	C20—C18—H18B	109.8
C7—C2—C1	109.8 (2)	H18A—C18—H18B	108.3
C2—C3—C4	109.0 (2)	C15—C19—C20	109.8 (2)
C2—C3—H3A	109.9	C15—C19—H19A	109.7
C4—C3—H3A	109.9	C20—C19—H19A	109.7
C2—C3—H3B	109.9	C15—C19—H19B	109.7
C4—C3—H3B	109.9	C20—C19—H19B	109.7
H3A—C3—H3B	108.3	H19A—C19—H19B	108.2
C10—C4—C5	109.6 (2)	C19—C20—C13	109.7 (2)
C10—C4—C3	109.9 (2)	C19—C20—C18	109.1 (2)
C5—C4—C3	108.8 (2)	C13—C20—C18	109.9 (2)
C10—C4—H4A	109.5	C19—C20—H20A	109.4
C5—C4—H4A	109.5	C13—C20—H20A	109.4
C3—C4—H4A	109.5	C18—C20—H20A	109.4
C6—C5—C4	110.0 (2)	C22—C21—H21A	109.5
C6—C5—H5A	109.7	C22—C21—H21B	109.5
C4—C5—H5A	109.7	H21A—C21—H21B	109.5
C6—C5—H5B	109.7	C22—C21—H21C	109.5
C4—C5—H5B	109.7	H21A—C21—H21C	109.5
H5A—C5—H5B	108.2	H21B—C21—H21C	109.5
C9—C6—C5	109.7 (2)	C23—C22—C27	117.6 (2)
C9—C6—C1	109.4 (2)	C23—C22—C21	121.3 (3)
C5—C6—C1	109.2 (2)	C27—C22—C21	121.1 (3)
C9—C6—H6A	109.5	C22—C23—C24	121.6 (2)
C5—C6—H6A	109.5	C22—C23—H23A	119.2
C1—C6—H6A	109.5	C24—C23—H23A	119.2
C2—C7—C8	109.01 (19)	C25—C24—C23	119.7 (2)
C2—C7—H7A	109.9	C25—C24—H24A	120.1
C8—C7—H7A	109.9	C23—C24—H24A	120.1
C2—C7—H7B	109.9	C24—C25—C26	119.5 (2)
C8—C7—H7B	109.9	C24—C25—S1	121.00 (18)
H7A—C7—H7B	108.3	C26—C25—S1	119.45 (18)
C7—C8—C10	109.6 (2)	C27—C26—C25	119.7 (2)
C7—C8—C9	109.6 (2)	C27—C26—H26A	120.1
C10—C8—C9	109.2 (2)	C25—C26—H26A	120.1
C7—C8—H8A	109.5	C26—C27—C22	121.9 (2)
C10—C8—H8A	109.5	C26—C27—H27A	119.1
C9—C8—H8A	109.5	C22—C27—H27A	119.1
C6—C9—C8	109.5 (2)	C29—C28—H28A	109.5
C6—C9—H9A	109.8	C29—C28—H28B	109.5
C8—C9—H9A	109.8	H28A—C28—H28B	109.5
C6—C9—H9B	109.8	C29—C28—H28C	109.5
C8—C9—H9B	109.8	H28A—C28—H28C	109.5
H9A—C9—H9B	108.2	H28B—C28—H28C	109.5
C4—C10—C8	109.3 (2)	C30—C29—C34	117.6 (2)
C4—C10—H10A	109.8	C30—C29—C28	120.6 (3)
C8—C10—H10A	109.8	C34—C29—C28	121.7 (3)
C4—C10—H10B	109.8	C29—C30—C31	121.9 (2)
C8—C10—H10B	109.8	C29—C30—H30A	119.1
H10A—C10—H10B	108.3	C31—C30—H30A	119.1
N1—C11—C12	108.65 (18)	C32—C31—C30	119.6 (2)
N1—C11—C14	108.76 (18)	C32—C31—H31A	120.2
C12—C11—C14	109.98 (18)	C30—C31—H31A	120.2
N1—C11—C13	109.11 (18)	C31—C32—C33	119.3 (2)
C12—C11—C13	110.30 (18)	C31—C32—S2	120.51 (18)
C14—C11—C13	110.00 (18)	C33—C32—S2	120.14 (19)
C11—C12—C17	109.03 (18)	C34—C33—C32	120.2 (2)
C11—C12—H12A	109.9	C34—C33—H33A	119.9
C17—C12—H12A	109.9	C32—C33—H33A	119.9
C11—C12—H12B	109.9	C33—C34—C29	121.4 (2)
C17—C12—H12B	109.9	C33—C34—H34A	119.3
H12A—C12—H12B	108.3	C29—C34—H34A	119.3
			
C6—C1—C2—N2	179.9 (2)	C11—C12—C17—C16	60.3 (2)
C6—C1—C2—C3	−60.5 (3)	C16—C17—C18—C20	−60.1 (3)
C6—C1—C2—C7	60.8 (3)	C12—C17—C18—C20	59.5 (3)
N2—C2—C3—C4	−179.4 (2)	C16—C15—C19—C20	60.1 (3)
C7—C2—C3—C4	−60.1 (3)	C14—C15—C19—C20	−59.9 (3)
C1—C2—C3—C4	61.0 (3)	C15—C19—C20—C13	60.2 (3)
C2—C3—C4—C10	59.7 (3)	C15—C19—C20—C18	−60.3 (3)
C2—C3—C4—C5	−60.4 (3)	C11—C13—C20—C19	−59.8 (2)
C10—C4—C5—C6	−59.5 (3)	C11—C13—C20—C18	60.2 (3)
C3—C4—C5—C6	60.7 (3)	C17—C18—C20—C19	60.1 (3)
C4—C5—C6—C9	59.4 (3)	C17—C18—C20—C13	−60.2 (3)
C4—C5—C6—C1	−60.5 (3)	C27—C22—C23—C24	0.7 (4)
C2—C1—C6—C9	−60.4 (3)	C21—C22—C23—C24	−179.0 (3)
C2—C1—C6—C5	59.7 (3)	C22—C23—C24—C25	−0.3 (4)
N2—C2—C7—C8	−180.0 (2)	C23—C24—C25—C26	−0.5 (4)
C3—C2—C7—C8	60.5 (3)	C23—C24—C25—S1	176.9 (2)
C1—C2—C7—C8	−60.7 (3)	O3—S1—C25—C24	8.2 (2)
C2—C7—C8—C10	−60.0 (3)	O2—S1—C25—C24	−112.6 (2)
C2—C7—C8—C9	59.8 (3)	O1—S1—C25—C24	129.4 (2)
C5—C6—C9—C8	−59.7 (3)	O3—S1—C25—C26	−174.4 (2)
C1—C6—C9—C8	60.1 (3)	O2—S1—C25—C26	64.8 (2)
C7—C8—C9—C6	−60.0 (3)	O1—S1—C25—C26	−53.2 (2)
C10—C8—C9—C6	60.1 (3)	C24—C25—C26—C27	0.9 (4)
C5—C4—C10—C8	59.9 (3)	S1—C25—C26—C27	−176.5 (2)
C3—C4—C10—C8	−59.7 (3)	C25—C26—C27—C22	−0.5 (4)
C7—C8—C10—C4	59.9 (3)	C23—C22—C27—C26	−0.3 (4)
C9—C8—C10—C4	−60.1 (3)	C21—C22—C27—C26	179.4 (3)
N1—C11—C12—C17	−179.86 (19)	C34—C29—C30—C31	−1.1 (4)
C14—C11—C12—C17	−60.9 (2)	C28—C29—C30—C31	178.7 (3)
C13—C11—C12—C17	60.6 (2)	C29—C30—C31—C32	−0.6 (4)
N1—C11—C13—C20	179.99 (19)	C30—C31—C32—C33	1.6 (4)
C12—C11—C13—C20	−60.7 (2)	C30—C31—C32—S2	−175.92 (18)
C14—C11—C13—C20	60.8 (2)	O5—S2—C32—C31	99.4 (2)
N1—C11—C14—C15	179.24 (19)	O4—S2—C32—C31	−138.5 (2)
C12—C11—C14—C15	60.4 (2)	O6—S2—C32—C31	−19.9 (2)
C13—C11—C14—C15	−61.3 (2)	O5—S2—C32—C33	−78.1 (2)
C11—C14—C15—C16	−59.6 (3)	O4—S2—C32—C33	44.0 (2)
C11—C14—C15—C19	60.2 (2)	O6—S2—C32—C33	162.6 (2)
C19—C15—C16—C17	−59.6 (3)	C31—C32—C33—C34	−1.0 (4)
C14—C15—C16—C17	59.9 (3)	S2—C32—C33—C34	176.6 (2)
C15—C16—C17—C18	59.9 (3)	C32—C33—C34—C29	−0.7 (4)
C15—C16—C17—C12	−60.0 (3)	C30—C29—C34—C33	1.7 (4)
C11—C12—C17—C18	−59.6 (3)	C28—C29—C34—C33	−178.0 (3)

### Hydrogen-bond geometry (Å, °)

**Table:**

Cg9 and Cg10 are the centroids of the C22–C27 and C29–C34 rings, respectively.

**Table:**

*D*—H···*A*	*D*—H	H···*A*	*D*···*A*	*D*—H···*A*
N1—H1C···O4	0.89 (2)	2.02 (2)	2.908 (3)	177 (3)
N1—H1D···O5^i^	0.90 (2)	1.99 (2)	2.883 (3)	177 (3)
N1—H1E···O6^ii^	0.89 (2)	1.92 (2)	2.806 (3)	173 (3)
N2—H2C···O1	0.91 (2)	1.93 (2)	2.834 (3)	174 (3)
N2—H2B···O2^iii^	0.89 (2)	1.92 (2)	2.806 (3)	170 (3)
N2—H2A···O3^iv^	0.89 (2)	2.01 (2)	2.901 (3)	175 (3)
C4—H4A···Cg10^iv^	0.98	3.18	3.878 (3)	130
C7—H7B···Cg9^iii^	0.97	2.87	3.801 (3)	161
C19—H19B···Cg10^v^	0.97	2.91	3.861 (3)	167

Symmetry codes: (i) −*x*+1, −*y*+1, −*z*+2; (ii) −*x*+2, −*y*+1, −*z*+2; (iii) −*x*+1, −*y*+1, −*z*+1; (iv) *x*−1, *y*, *z*; (v) *x*, *y*−1, *z*.
